# Prognostic factors and long term results of neo adjuvant therapy followed by surgery in stage IIIA N2 non-small cell lung cancer patients

**DOI:** 10.4103/1817-1737.56010

**Published:** 2009

**Authors:** Jing Li, Chun-Hua Dai, Shun-Bing Shi, Ping Chen, Li-Chao Yu, Jian-Rong Wu

**Affiliations:** *Department of Pulmonary Medicine, Affiliated Hospital of Jiangsu University, Zhenjiang, Jiangsu, China*; 1 *Department of Radiation Oncology, Affiliated Hospital of Jiangsu University, Zhenjiang, Jiangsu, China*; 2 *Department of Thoracic Surgery, Affiliated Hospital of Jiangsu University, Zhenjiang, Jiangsu, China*; 3 *Department of Pathology, Affiliated Hospital of Jiangsu University, Zhenjiang, Jiangsu, China*

**Keywords:** Neo adjuvant therapy, non-small cell lung cancer, prognostic factor, stage IIIA, surgery, survival

## Abstract

**BACKGROUND:**

Prognosis of stage IIIA N2 non-small cell lung cancer (NSCLC) remains poor despite the changes in therapeutic strategies.

**OBJECTIVES:**

To assess long term results of neo adjuvant therapy followed by surgery for patients with stage IIIA N2 NSCLC and to analyze factors influencing survival.

**MATERIALS AND METHODS:**

The methods adopted include: Retrospective review of medical records of 91 patients with stage IIIA N2 NSCLC, who received neo adjuvant therapy followed by surgery; collection of information on demographic information, staging procedure, preoperative therapy, clinical response, type of resection, pathologic response of tumor, status of lymph nodes and adjuvant chemotherapy; survival analysis by Kaplan-Meier and calculation of prognostic factors using log-rank and Cox regression model.

**RESULTS:**

All patients received a platinum-based chemotherapy and 23 (29.1%) had an associated radiotherapy. Eighty four patients underwent thoracotomy. Median survival was 26 months (95%CI, 22.6-30.8 months) with three and five year survival rates of 31.6 and 20.9%, respectively. Prognostic factors for survival on univariate analysis was clinical response (*P*= 0.032), complete resection (*P*= 0.002), pathologic tumor response (*P*< 0.001), and lymph nodal down staging (*P* = 0.001). Multivariate analyses identified complete resection, pathologic tumor response and lymph nodal down staging as independent prognostic factors.

**CONCLUSION:**

Survival of patients with stage IIIA N2 NSCLC who received neo adjuvant therapy is significantly influenced by clinical response, complete resection, pathologic tumor response, and lymph nodal down staging. These results can be helpful in guiding standard clinical practice and evaluating the outcome of neo adjuvant therapy followed by surgery in patients with stage IIIA N2 NSCLC.

Stage IIIA N2 non-small cell lung cancer (NSCLC) with involvement of ipsilateral mediastinal N2 represents a heterogeneous group of patients. For patients with this locally advanced disease, five-year survival rate after surgery or radiotherapy alone is only approximately 10%; majority of the patients died of distant metastases.[1,2] This observation led to attempts to improve survival by adopting combined modality approaches such as neo adjuvant chemotherapy followed by surgery and/or radiotherapy. The rationale for using neo adjuvant chemotherapy for the treatment of stage IIIA NSCLC is based on evidence that chemotherapy can reduce tumor burden which could facilitate surgery, improve resectability and eradicate micrometastases to prevent systemic relapse.[3,4] Several phase II trials show the role of neo adjuvant chemotherapy in patients with locally advanced NSCLC.[5–7] Two small randomized trials suggest a prolonged survival for neo adjuvant chemotherapy followed by surgery compared with surgery alone for stage IIIA NSCLC.[8–11] However, a phase III randomized trial reported by Nagai *et al*. failed to demonstrate any benefit of neo adjuvant chemotherapy followed by surgery over surgery alone.[12] In another phase III trial in resectable stages I (except T1 N0), II and IIIA NSCLC patients, neo adjuvant chemotherapy failed to improve survival of stage IIIA N2 patients.[13] Inconsistency of results from randomized trials assessing neo adjuvant chemotherapy for stage IIIA NSCLC is due to small sample sizes, short follow-up periods, and unacceptable toxicity for certain of the chemotherapy regimens administered. To date, management of stage IIIA N2 NSCLC is controversial, and there is no agreement about the best approach to patients with involvement of ipsilateral mediastinal lymph node. Because of the demonstrated heterogeneity in this patient population,[14] it is not yet clear for which subset of these patients this therapeutic strategy (neo adjuvant therapy followed by surgery) will be most rewarding. The identification of prognostic factors could be useful for better selection of therapy and design of future randomized trials.

In this study, we review our experience between 1998 and 2004, to analyze prognostic factors and better define the long-term survival of Chinese patients with stage IIIA NSCLC treated with neo adjuvant therapy followed by surgery.

## Materials and Methods

### Patients

Medical records of all patients with stage IIIA N2 NSCLC who underwent surgery following neo adjuvant therapy at our hospital were reviewed retrospectively for the period from January 1998 to October 2004. We limited our analyses to patients who had been clinically staged as IIIA N2 disease and received platinum-based neo adjuvant chemotherapy (with or without radiotherapy) including patients who underwent thoracotomy, had a clinical response and stable disease as well as patients who were not operated upon due to progressive disease. Hence the patients who achieved surgical resection had completely postoperative pathologic information. Data recorded included demographic information, staging procedures and pretreatment clinical stage. All patients were staged on the basis of history, physical examination, chest X-ray, bronchoscopy, brain and chest computed tomography (CT) scan, abdominal ultrasound or abdominal CT scan, radionuclide bone scan. Tumors were staged according to the revised version of TNM staging published in August 1997.[15]

Criteria for pretherapy diagnosis of stage IIIA NSCLC with clinical N2 (cN2) were defined as a mediastinal node of short axis diameter exceeding 15mm on CT scan. Cases with a diameter between 10 and 14mm were included only of there was more than one node. Patients with N3 disease revealed by CT scan were excluded from this study. This study was approved by the Ethics Committee of Affiliated Hospital of Jiangsu University in China.

### Patient treatment and assessment

Data on chemotherapy regimen, number of cycles, radiotherapy and clinical response to neo adjuvant therapy, surgical procedure and site of the first cancer recurrence post-operation was collected. After completion of neo adjuvant therapy, chest CT scan was repeated for all patients to assess the clinical response to therapy and feasibility for surgery. Brain CT and bone scan were repeated if clinically indicated. Patient's responses to chemotherapy were categorized as complete, partial response, stable or progressive disease according to World Health Organization (WHO) criteria.

Patients who had a clinical response and stable disease received thoracotomy. The extent of the performed pulmonary resection was left to the discretion of the attending surgeon based on the intraoperative assessment of the residual disease. All accessible hilar lymph nodes were dissected from the specimen, and a complete mediastinal lymph node dissection was performed for all patients. A complete resection (R0) was defined as pathologic demonstration of negative tissue margins and all detectable disease had been removed. Patients who had a complete gross resection, in whom positive margins were found on final pathologic review, were classified as microscopic incomplete resection (R1). Gross residual disease after surgical resection was classified as macroscopic incomplete resection (R2). Pathologic response, status of lymph node, operative morbidity and mortality and postoperative treatment when appropriate were recorded.

Pathologic response of primary tumor was examined using criteria reported by de Bore *et al.*[16] Pathologic complete response (pCR) was defined as absence of any tumor in the surgical specimen; partial response (pPR) was defined as the presence of only small residual foci of tumor cells, or the presence of significant areas of tumor necrosis throughout the surgical specimen; no change (pNC) was defined as the presence of large area of identifiable tumor cells in the surgical specimen with minimal evidence of tumor necrosis. Nodal status classification was determined from a postoperative pathologic examination. Down staging was defined as complete fibrosis, scar, or granulation of lymph nodes, with neither normal nodal formation nor cancer cell in the surgical specimen. Patients with normal nodal formation in ipsilateral mediastinum were excluded from the study. Operative morbidity was defined as any event occurring during the first 30 days following surgery.

### Statistical analysis

Overall survival was calculated from the first cycle of neo adjuvant chemotherapy to the date of death or date of last follow-up. Survival data was last collected in May 2008. Survival curve were estimated using the Kaplan-Meier method. The following variables were considered potentially prognostic variables for survival: Sex, initial tumor stage, histology, chemotherapy regimen, adjunction of radiotherapy, clinical response to neo adjuvant therapy, type of surgery, extent of surgical resection, pathologic tumor response, status of lymph node, and adjuvant chemotherapy.

Log-rank analysis was used for univariate analysis for significant prognostic factor. Cox regression model was used for the multivariable analysis of independent prognostic factor. Statistical analysis was performed using the SPSS V10.0 software package (SPSS Inc., Chicage, Illinois).

## Results

### Patient characteristics

From January 1998 to October 2004, 105 patients received neo adjuvant therapy for stage IIIA N2 NSCLC at our hospital. Of these, 91 had detailed clinical and follow-up data and were assessable and eligible for this study. The remaining patients were not assessable for the following reasons: No available data due to loss to follow-up (n=six), use of nonplatinum chemotherapy regime (n=four), patient refusal for surgery after neo adjuvant therapy (n=four). The patient characteristics and treatment information are listed in Table 1. There were 66 men and 25 women in the group. The median age was 59 years (range 38-70 years). Thirteen, 40, and 38 cases were classified as T1N2M0, T2N2M0, and T3N2M0, respectively. Forty two and 49 cases were identified as single and multiple lymph node involvement, respectively, according to pretherapy CT scan. Examination of bronchoscopic biopsy and brushing (73 patients) or percutaneous needle aspiration specimens (18 patients) were performed to determine the histologic types. Forty four patients had adenocarcinoma, 40 patients had squamous cell carcinoma, four had large cell carcinoma, and three had undifferentiated NSCLC.

**Table 1:** Patient characteristics and treatment

Characteristic	No. of patients (n = 91)	%
Sex		
Male	66	72.5
Female	25	27.5
Age (yr)		
Median	59	
Range	38-70	
Initial tumor stage		
T1N2M0	13	14.3
T2N2M0	40	44
T3N2M0	38	41.7
Histology		
Squamous cell carcinoma	40	44
Adenocarcinoma	44	48.4
Large cell carcinoma	4	4.4
Undifferentiated NSCLC	3	3.2
Chemotherapy regimen		
EP	12	13.2
NP	45	49.5
GP	34	37.3
Thoracic radiotherapy		
Yes	29	31.9
No	62	68.1
Clinical response		
Complete response	6	6.6
Partial response	36	39.6
Stable disease	42	46.1
Progressive disease	7	7.7
Type of operation (n = 84)		
Lobectomy	45	53.6
Biolobectomy	15	17.8
Pneumonectomy	24	28.6
Pathologic response of tumor (n = 84)		
Complete response	8	9.5
Partial response	41	48.8
No change	35	41.7
Pathologic lymph node status (n = 84)		
N2	56	66.7
N1	18	21.4
N0	10	11.9

EP-Etoposide and cisplatin; NP-Vinorelbine and cisplatin; GP-Gemcitabine and cisplatin

### Neo adjuvant treatment

The neo adjuvant chemotherapy regimen consisted of cisplatin (80 mg/m^2^ on day 1) plus etoposide (100 mg/m^2^ on days 1 to 5) in 12 patients (13.2%), cisplatin (80 mg/m^2^ on day 1) plus vinorelbine (30 mg/m^2^ on days 1 and 8) in 45 patients (49.5%) and cisplatin (80 mg/m^2^ on day 1) plus gemcitabine (1000 mg/m^2^ on days 1 and 8) in 34 patients (37.3%). Three patients received one cycle of chemotherapy, 76 received two cycles of chemotherapy, 12 received three cycles of chemotherapy. Twenty nine patients (31.9%) with N2-bulky disease, defined as multiple lymph node involvement on a CT scan with a minimal greatest dimension of two cm and with radiologic signs of extra capsular extension, underwent preoperative sequential (n = 13) or concurrent (n = 16) chemoradiotherapy. The sequential chemotherapy consisted of two cycles of chemotherapy with cisplatin plus etoposide or cisplatin plus vinorelbine at a 21-day treatment cycles. The following radiotherapy commenced three weeks after the start of the second cycles of chemotherapy with a total irradiation dose ranging from 40 to 50 Gy (a dose of 2.0 Gy administered daily, five days per week). The concurrent chemoradiotherapy consisted of two cycles of cisplatin (30 mg/m^2^ on days 1, 8, and 15) plus etoposide (50 mg/m^2^ on days 1 to 5) with concurrent, continuous chest radiotherapy, which began on day 1 of chemotherapy with a dose of 1.8 or 2.0 Gy daily, five days per week at the total dose ranging from 40 to 45 Gy.

The radiotherapy field included the primary tumor with 1.5 cm margin, the ipsilateral pulmonary hilum, upper and median mediastinal lymph nodes. Clinical response to neo adjuvant therapy is summarized in Table 1. Six patients (6.6%) achieved a complete response, 36 patients (39.6%) obtained a partial response, and 42 patients (46.2%) had stable disease, and seven patients (7.7%) had progressive disease. The overall response rate was 46.2%.

### Surgical treatment

Eighty four patients who had a clinical response and stable disease underwent thoracotomy. Seven patients experienced progressive disease received radiotherapy and further chemotherapy. Type of surgical resection included lobectomy (n = 45), bilobectomy (n = 15), and pneumonectomy (n = 24). A complete resection (R0) was achieved in 63 patients (75%), an R1 in 15 patients (17.9%), and an R2 in six patients (7.1%). The operative mortality rate was 2.4% (2/84). Thirteen patients (15.5%) had a non-lethal postoperative complication that included arrhythmia (n is equal to three), pneumonia (n is equal to three), bronchopleural fistula (n is equal to two), heart failure (n is equal to two), prolonged air leak (n is equal to two), and chylothorax (n is equal to one). Pathologic complete response of primary tumor (pCR) was found in eight patients (9.5%), including five patients with squamous cell carcinoma and three patients with adenocarcinoma, pPR was seen in 41 patients (48.8%), pNC was recorded in 35 patients (41.7%). pathologic responses were not entirely consistent with clinical response. Eighteen patients were down staged to N1, 10 patients were down staged to N0, and 56 patients remained N2 positive at operation. Down staging was seen in 20 (48%) of the 42 patients with single lymph node involvement and eight (16%) of 49 patients with multiple lymph node involvement. The proportion of down staging in patients with single lymph node involvement was significantly higher than patients with multiple lymph node involvement (*P* = 0.019). Fifteen patients had subcarinal lymph node involvement. Postoperative radiotherapy was administered on 17 out of 21 patients who either had an incomplete resection (R1 or R2) or involvement of the uppermost mediastinal lymph node. Thirty two patients (38.1%) received two to three cycles of adjuvant chemotherapy with cisplatin plus vinorelbine or cisplatin plus gemcitabine.

### Survival

The median follow-up time was 43 months. The median survival time from the date of starting neo adjuvant chemotherapy for all patients in this study was 26 months (95% CI, 21.6-30.8 months), and the one-, three-, and five-year survival rates was 86.1%, 31.6% and 20.9%, respectively. Forty seven (74.6%) of 63 patients who achieved a complete resection were known to have experienced a recurrence. The recurrence was loco regional in 16 patients (34%), distant in 24 patients (51.1%), and both loco regional and distant in seven patients (14.9%). Sites of distant metastases included brain (n is equal to 12), bone (n is equal to 10), liver (n is equal to five), adrenal glands (n is equal to two) and contra lateral lung (n is equal to two).

**Figure 1:**
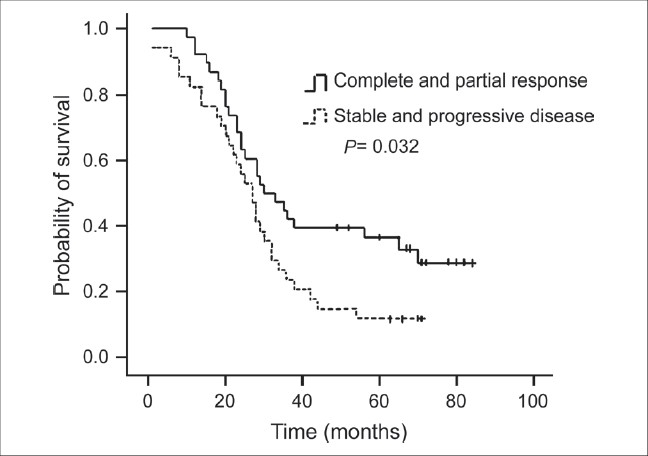
Kaplan-Meier survival curve according to respone to neo adjuvant chemotherapy; (Complete response and partial response versus stable and progressive disease, log rank test: *P* = 0.032)

**Figure 2:**
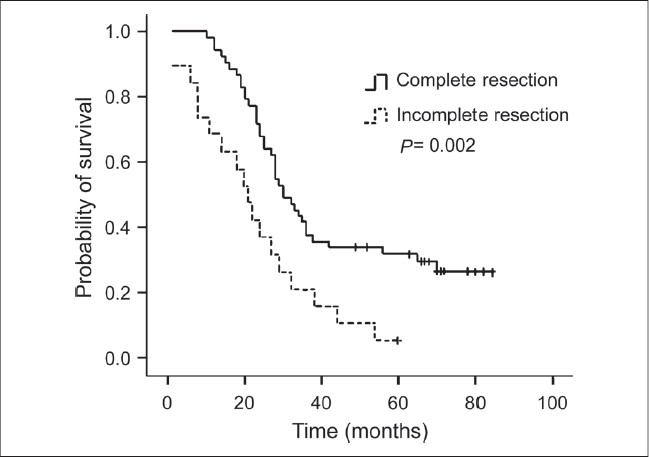
Kaplan-Meier survival curve according to extent of surgical resection; (Complete resection versus incomplete resection, log rank test: *P* = 0.002)

**Figure 3:**
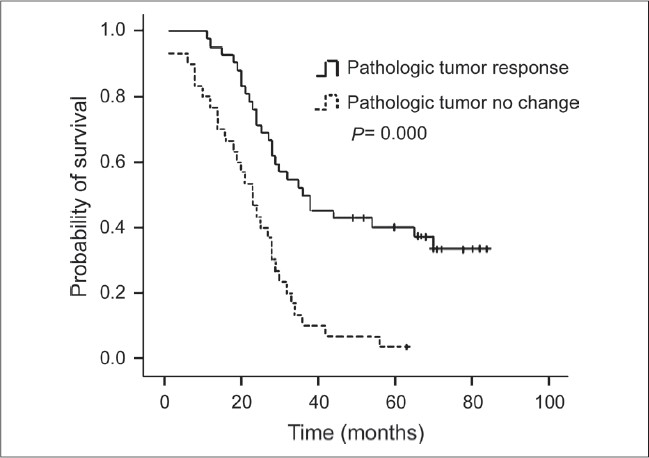
Kaplan-Meier survival curve according to pathologic response; (Pathologic complete response and partial response versus pathologic no change, log rank test: *P* < 0.001)

**Figure 4:**
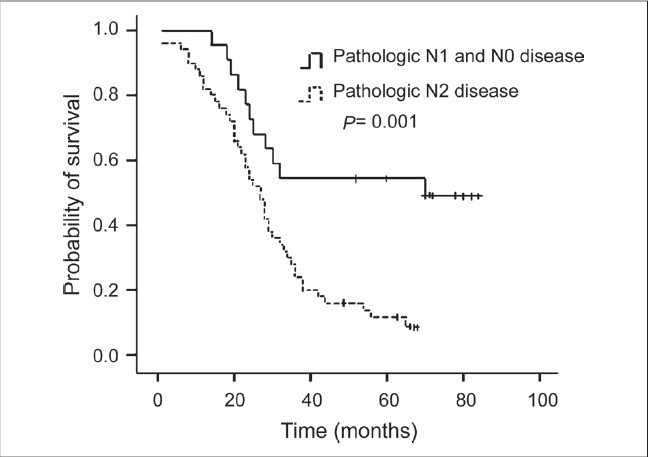
Kaplan-Meier survival curve according to lymph nodular status; (N0 or N1 disease versus N2 disease, log rank test: *P* = 0.001)

For the purposes of analysis, the squamous cell carcinoma patients were considered as one group, and the other patients who predominantly had ademocarcinoma were considered nonsquamous. The results of univariate analysis of prognostic factors for survival are shown in Table 2. Factors correlated with improved survival were clinical response (PR and CR), complete resection (R0), pathologic response of tumor (pCR and pPR), and lymph nodal down staging (N1 and N0). Patients who had clinical complete or partial response to neo adjuvant therapy had a 30-month median survival compared with 23 months for patients with stable disease and progressive disease [*P* = 0.032, Figure 1]. The patients with complete resection had a median survival of 30 months compared with 21 months for patients with incomplete resection [*P* = 0.002, Figure 2]. Pathologic complete and partial response was associated with 36 months median survival, compared with 23 months in patients with pathologic no change in surgical specimens [*P* < 0.001, Figure 3]. The median survival for patients with N1 and N0 disease was 37 months compared with 27 months for patients with residual N2 disease [*P* = 0.001, Figure 4]. Survival was not influenced by sex, initial clinical stage, histology, chemotherapy regimen, adjunction of radiotherapy, type of resection, and postoperative chemotherapy. Factors that demonstrated prognostic significance by univariate analysis were then examined in a multivariate analysis. Analysis of Cox regression model showed that extent of surgical resection (*P* = 0.001), pathologic response of tumor (*P* < 0.001), and lymph nodal down staging (*P* = 0.048) were independent prognostic factors [Table 3].

**Table 2:** Univariate analysis of factors influencing survival by log-rank test

Variables	Overall survival				*P*
		
	Median (months)	1-year (%)	3-years (%)	5-years (%)
Sex					
Male	28	86.3	31.4	20.9	0.593
Female	30	90.5	33.3	28.6	
Initial clinical stage					
T1-2 N2	32	88.9	44.4	29.2	0.324
T3 N2	27	86.7	26.7	22.0	
Histology					
Squamous cell carcinoma	29	87.9	36.4	23.9	0.772
Nonsquamous cell carcinoma	28	84.6	35.9	22.2	
Radiotherapy					
Yes	30	86.4	35.3	29.4	0.164
No	28	81.1	27.7	12.4	
Clinical response					
CR and PR	30	92.1	42.1	36.4	0.032
SD and PD	23	71.4	19.2	9.5	
Type of surgery					
Pneumonectomy	25	84.2	26.3	10.5	0.129
Lobectomy and bilobectomy	29	88.7	35.8	28.1	
Extent of resection					
Complete resection	30	94.3	41.5	31.8	0.002
Incomplete resection	21	68.4	21.1	0	
Pathologic response					
pCR and pPR	36	95.2	50.0	40.2	<0.001
pNC	23	76.7	10.0	0	
Lymph nodal downstaging					
N0 and N1	37	95.5	54.5	49.1	0.001
N2	27	82.0	28.0	11.4		
Adjuvent chemotherapy					
Yes	29	96.2	38.5	18.5	0.912
No	27	82.6	34.8	28.1	

CR-Complete response; PR-Partial response; SD-Stable disease; PD-Progressive disease; pCR-Pathologic complete response; pPR-Pathologic partial response; pNC- Pathologic no change

**Table 3:** Multivariate analysis of prognostic factors by Cox regression model

Variables	Hazard radio	95%CI	*P*
Clinical response	1.095	0.824-1.372	0.367
(CR and PR versus SD and PD)			
Extent of resection	2.869	1.556-5.288	0.001
(Complete versus incomplete resection)			
Pathologic tumor response	3.295	1.827-5.943	<0.001
(pCR and pPR versus pNC)			
Lymph nodal downstaging	2.074	1.007-4.267	0.048
(N1 and N0 versus N2)			

CI-Confidence interval; CR-Complete response; PR-Partial response; SD-Stable disease; PD-Progressive disease; pCR-Pathologic complete response; pPR-Pathologic partial response; pNC-Pathologic no change

## Discussion

Although neo adjuvant chemotherapy is considered a standard of treatment in patients with resectable stage IIIA NSCLC in the updated guideline of the European Society of Medical Oncology,[17] there are still many controversial opinions on the optimal approach of locally advanced N2 disease. During the last 10-15 years, many phase II trials had suggested that neo adjuvant chemotherapy with or without radiotherapy followed by surgery was feasible and tolerable, and can improve surgical resectability and survival for stage IIIA N2 NSCLC.[5–7,18,19] Moreover, the response rate to neo adjuvant chemotherapy in patients with stage III and early stage disease as well as good performance status were significantly higher than those patients with advanced stage IV NSCLC.[18,19] However, the significant variability in designs of these trials resulted in discrepancy of results and variable survival data reported. Recent studies have analyzed prognostic factors for long-term survival in patients with locally advanced NSCLC underwent surgery following neo adjuvant therapy.[19–23] In the present study, we examined the prognostic factors and the outcome of patients with stage IIIA N2 NSCLC and treated with neo adjuvant chemotherapy with or without radiotherapy followed by surgery. In univariate analysis, survival was significantly influenced by the clinical response (*P* = 0.032) or surgical complete resection (*P* = 0.002). These results are consistent with trials reported by Lorent *et al*.[19] and Betticher *et al*.[20] The study by Lorent *et al*. showed that patients who achieved complete resection after neo adjuvant chemotherapy had an median survival of 58 months, with five-year survival rate of 38.6%, compared with 17 months and zero per cent, respectively, in those patients who had incomplete resection (*P* = 0.004). Pathologic tumor response (*P* < 0.001) and lymph nodal down staging (*P* = 0.001) had also been identified as strong predictors of survival on our univariate analysis.

Further, we found that patients with single lymph node involvement had significantly higher rate of down staging when compared with patients with multiple lymph nodes, which may be due to less tumor burden in the former. A study by Thomas *et al*.[21] demonstrated that the degree of tumor regression examined in the resection specimens was predictive for survival, the median survival of 36 months versus 14 months for tumor regression >90% compared with tumor regression less than or equal to 90% patients, and three-year survival rates of 48% versus nine per cene (*P* = 0.02). The SWOG study[22] showed 30-month median survival for patients with N0 and N1 disease compared with 10 months for N2 disease with a three-year survival rate of 44 versus 18% (*P* < 0.001). Though these trials included patients with stage IIIB disease and thoracic radiotherapy was given, it indicated that the degree of pathologic response and lymph nodal down staging can significantly influence survival in patients with locally advanced NSCLC after neo adjuvant treatment.

The female sex and early tumor stage (smaller tumor size) have been associated with better survival prognosis in previous studies[23,24] but not in our analysis. Betticher *et al*.[20] in a trial show that histology (squamous cell carcinoma) correlated with a better pathologic response to neo adjuvant chemotherapy while the impact of histology on survival prognosis remains undefined. The same results were found in our study. Just like some reports analyzing predictive prognosis factors to neo adjuvant therapy,[23,25] we failed to find substantial difference in survival rate of patients using combined radiotherapy and chemotherapy as compared with those using neo adjuvant chemotherapy alone. It is possible that the addition of radiotherapy to neo adjuvant chemotherapy may not affect survival because it eradicates loco regional tumor without treating systemic disease, which is the main cause of death in this patient population. Some investigators reported that pneumonectomy was significantly associated with a shorter survival when compared with other types of resection (lobectomy and bilobectomy).[23,25] However, we did not confirm a significant long-term adverse influence of pneumonectomy. The reason for this discrepancy could be patient selection for performing pneumonectomy and small sample size.

Our multivariate analysis shows that pathologic tumor response after neo adjuvant treatment is the most powerful prognostic factor for survival (*P* < 0.001). A second strong prognostic factor for survival is complete resection (*P* = 0.001). Lymph nodal down staging is also an independent prognostic factor (*P* = 0.048). These results are similar to those reported in trials studying outcome of patients with locally advanced NSCLC after surgery following neo adjuvant treatment.[20,23,25] The data could suggest that absence of tumor in the mediastinal lymph nodes after neo adjuvant treatment may serve as a positive sign of the possible eradication of micrometastasis, while a significant pathologic response of primary tumor may well reflect the efficient local control. In addition, surgical resection is an efficient salvaging procedure for stage IIIA patients who had partial response and residual vital tumor tissue in resection specimens after neo adjuvant treatment.

Some investigators report that survival results of the patients received surgery are not significantly better than those of patients receiving radiotherapy or chemoradiotherapy following neo adjuvant chemotherapy.[26,27] Only part of patients with stage III NSCLC may be suitable candidates for surgery, thus pulmonary resection should be performed in carefully selected patients with stage III NSCLC after neo adjuvant therapy. On the other hand, it is possible that patients who have a pathologically good response to chemotherapy would be expected to have the best prognosis regardless of whether they undergo surgery or not. Several trials including our retrospective study have demonstrated that pathologic response to neo adjuvant chemotherapy or chemoradiotherapy is an independent prognostic factor influencing survival.[19,20]

In this study, we adopted the lymph node size-based criteria on CT scan for clinical staging. We have acknowledged no mediastinoscopy for staging prior to neo adjuvant therapy as a drawback with this article. In general, mediastinoscopy is routinely used as a part of the preoperative assessment in patients with locally advanced NSCLC.

Okada *et al*.[28] and Cappuzzo *et al*.[29] have published data on many patients with N2 disease who underwent surgery following neo adjuvant therapy without prior mediastinoscopy. They also used lymph node size on CT scan as staging criteria. Furthermore, in the present study, N2 status was determined finally according to the post-operative pathologic examination of mediastinal lymph nodes. Pathologic finding indicating complete fibrosis, scar, or granulation of lymph nodes, with neither normal nodal formation nor cancer cell, meant pathologic complete response of lymph nodes (down staging). Therefore, N2 status can be determined at least in patients who achieved surgical resection.

The most important factor to judge a study on neo adjuvant therapy is long-term survival. Our results confirm that clinical response, surgical complete resection, pathologic response of tumor and lymph nodal down staging after neo adjuvant therapy for stage IIIA NSCLC, are the prognostic factors identified as influencing survival. These results reinforce the current knowledge about the outcome of locally advanced NSCLC patients submitted to neo adjuvant therapy, and may help in guiding standard clinical practice.
